# Surveillance for lymphatic filariasis after stopping mass drug administration in endemic districts of Togo, 2010–2015

**DOI:** 10.1186/s13071-018-2843-3

**Published:** 2018-04-16

**Authors:** Monique Ameyo Dorkenoo, Rachel Bronzan, Degninou Yehadji, Mawèke Tchalim, Kossi Yakpa, Santrao Etassoli, Poukpessi Adjeloh, Issaka Maman, Yao Sodahlon

**Affiliations:** 1 Faculté des Sciences de la santé, Université de Lomé, BP 1515 Lomé, Togo; 2 Programme National d’Elimination de la Filariose Lymphatique, Ministère de la Santé et de la Protection Sociale, Angle avenue Sarakawa et Avenue du 24 Janvier, BP 336 Lomé, Togo; 3 Health and Development International (HDI), Newburyport, MA USA; 4 Programme National de Lutte contre le Paludisme, BP 518 Lomé, Togo; 5 Laboratoire de référence, Institut National d’Hygiène, BP 1396 Lomé, Togo; 6 Mectizan Donation Program, 325 Swanton Way, Decatur, GA 30030 USA

**Keywords:** Lymphatic filariasis, Mass drug administration, Transmission assessment survey, Post-treatment surveillance, Togo

## Abstract

**Background:**

Togo is a country previously endemic for lymphatic filariasis (LF). In 2010, following nine years of mass drug administration (MDA) for LF, the country established a post-treatment surveillance (PTS) system. We present here the results of these PTS activities, carried out from 2010 to 2015, as well as the findings of follow-up investigations in 2016 to confirm the absence of infection in previously infected individuals.

**Methods:**

The routine surveillance established in 2010 consisted of a network of 47 laboratories, which searched for *Wuchereria bancrofti* microfilaria on nocturnal blood smears collected for malaria diagnosis and an additional network of 20 peripheral health facilities, which collected dried blood spots and tested them for Og4C3 antigen. Two transmission assessment surveys (TAS) were also undertaken, as recommended by WHO, in 2012 and 2015. Any positive case identified through any surveillance activity was immediately retested by nocturnal smear and confirmed cases were immediately investigated by screening family members and neighboring household members. In 2016, 32 of the 40 positive cases detected during TAS or laboratory and health facility network activities were traced and whether confirmed positive by nocturnal smear or not were tested again simultaneously by filariasis test strip (FTS), Og4C3 and a nocturnal blood smear to rule out any active infection.

**Results:**

From 2010 to 2015, the laboratory network identified one microfilaria-positive individual (0.0% of 26,584 persons tested) and the peripheral health facility network detected 19 Og4C3-positive individuals (0.28% of 6788 persons tested). All 19 Og4C3 cases were negative for microfilaremia by nocturnal blood smear. In the 2012 and 2015 TAS, thirteen and six ICT/FTS positive cases, respectively, were identified, which were significantly below the critical cut-off (18–20 cases) across all evaluation units. Three of the six ICT/FTS-positive cases from the 2015 TAS were positive by nocturnal smear; immediate investigation identified one additional microfilaria-positive individual. Epidemiological investigation revealed that four of the five cases of microfilaremia were imported from another country in the region. In 2016, 32 of the 40 positive cases detected by at least one test during all surveillance activities were traced: four (12.5%) individuals were still positive by FTS but all 32 individuals were negative for microfilaremia and Og4C3 antigen.

**Conclusion:**

The results of post-treatment surveillance in Togo have demonstrated that *W. bancrofti* filariasis is no longer of public health concern in Togo, more than six years after stopping MDA. Every possible effort should be made to maintain surveillance in order to promptly detect any resurgence and preserve this achievement.

## Background

Lymphatic filariasis (LF) is a severely debilitating, disfiguring and stigmatizing mosquito-borne disease caused by infection with the nematode species *Wuchereria bancrofti*, *Brugia malayi* or *Brugia timori*. The parasites are transmitted by various species of mosquito vectors: *Anopheles*, *Aedes*, *Culex*, *Mansonia* and *Ochlerotatus* [1, 2]. Currently the second leading infectious cause of disability worldwide, LF is endemic in 73 countries, an estimated 120 million people are infected with the parasites and 40 million people suffer from complications such as hydrocele, lymphedema and elephantiasis [3]. LF is one of the neglected tropical diseases targeted by the World Health Organization (WHO) for global elimination by 2020 [4]. The elimination strategy has two components: (i) transmission interruption through drug administration to every eligible person in endemic areas; and (ii) morbidity management and prevention of disability by providing access to basic care for LF-related diseases to every affected person in endemic areas [5, 6].

Togo is one of the 34 African countries endemic for LF and is surrounded by three other endemic countries: Benin, Ghana and Burkina Faso. Based on the mapping of LF in 2000, eight of Togo’s 40 health districts (7 of 35 districts, prior to administrative restructuring in 2012) were identified as endemic [7]. After nine years of mass drug administration (MDA) with satisfactory outcomes per the WHO guidelines and based on evidence from a transmission assessment survey (TAS) of the probable interruption of transmission, Togo stopped mass treatment for LF in 2009 and initiated post-treatment surveillance (PTS) activities in 2010 [8].

The WHO recommends conducting TAS two to three and five to six years after stopping MDA, but does not make recommendations for additional PTS. In Togo, at the National LF Elimination Programme (NLFEP), we successfully carried out two TAS, in 2012 and 2015, three and six years, respectively, following the last MDA in 2009 [9, 10]. We additionally established an extensive PTS system with comprehensive geographical coverage that included epidemiological investigations of all microfilaria-positive individuals. In 2016, we retraced all individuals identified through the PTS system as having been infected with LF and retested them to determine their outcome and provide additional evidence of the effectiveness of the PTS system. This paper presents the results of these surveillance activities during the 2010–2015 period and the 2016 follow-up activity.

## Methods

### Ongoing post-treatment surveillance system

**Fig. 1:**
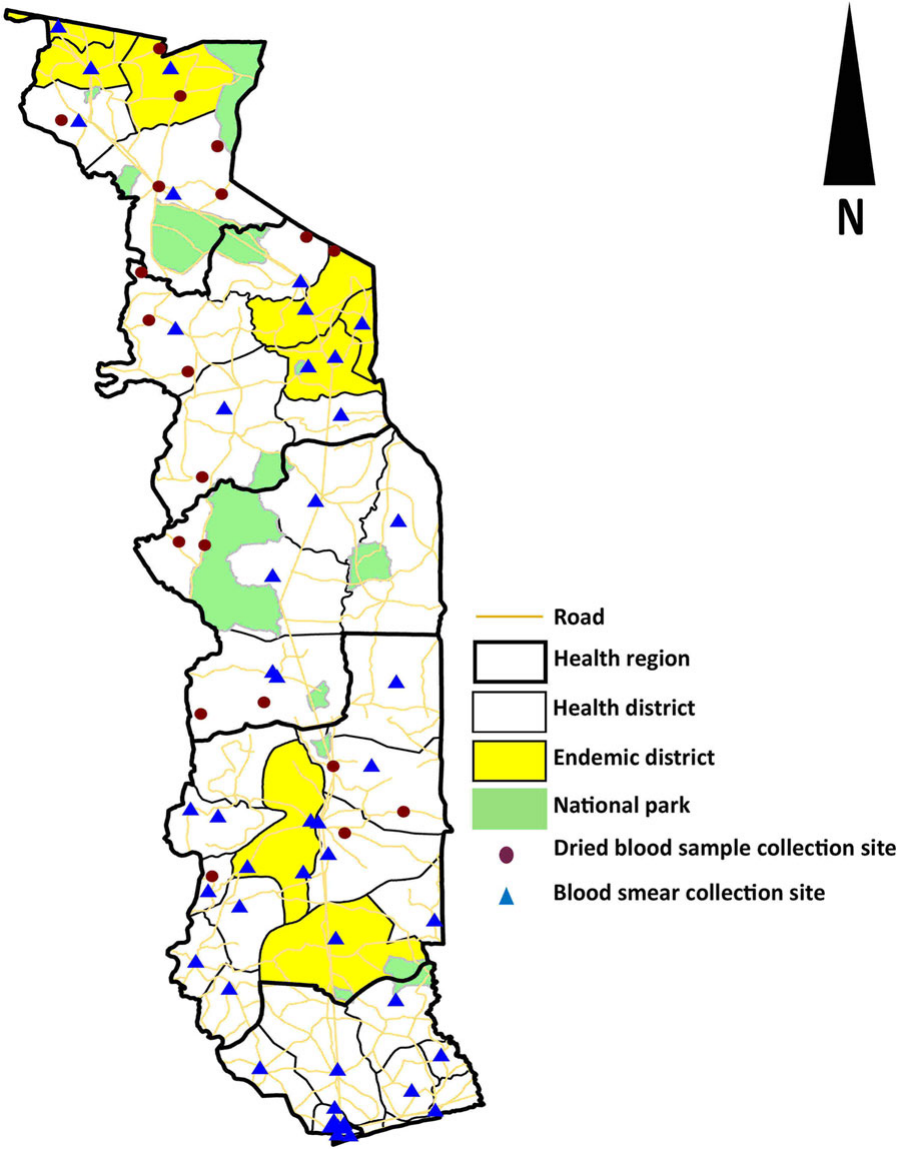
Blood smear and dried blood sample collection sites (laboratories and peripheral health centers) for passive LF surveillance in Togo

In 2006, before MDA was stopped, Togo established an innovative laboratory-based routine surveillance system to supplement the WHO-recommended PTS TAS. The system covered endemic as well as non-endemic districts and consisted of searching for *W. bancrofti* microfilaria on thick blood smears prepared for malaria diagnosis. An evaluation of this laboratory-based system carried out in 2010 showed that some areas of the country were not covered by the system. A complementary system was therefore set up to search for *W. bancrofti* antigen in dried blood samples (DBS) collected from patients in selected healthcare facilities in areas not covered by the laboratory surveillance system [9, 11]. Thus, the definitive ongoing surveillance system had two components (Fig. 1). The first component was a network of 47 laboratories (at least one lab per health district) in which the laboratory technicians routinely searched for microfilaria on all blood smears performed between 22:00 and 3:00 h for malaria diagnosis [11]. Every month, 10 randomly selected blood smears and any positive smear were shipped to the reference laboratory of the NLFEP in Lomé for quality control testing. Any microfilariae-positive smear supplied by a technician of the laboratory network was confirmed with a second nocturnal blood smear by technician of the NLFEP’s reference laboratory. All blood smears were stained with Giemsa.

The second component was a network of 20 peripheral healthcare facilities in areas not covered by the laboratory network. At four-month intervals, each of the managing nurses of these peripheral healthcare facilities collected dried blood samples from at least 20 patients of any age who agreed to participate. These samples were shipped to the immunology laboratory of the National Institute of Hygiene, Lomé (NIH), the national public health laboratory, to perform an enzyme-linked immunosorbent assay (ELISA) for detection of Og4C3 antigen specific to *W. bancrofti* microfilaria [10, 12, 13]. Individuals testing positive by Og4C3 ELISA were revisited and tested by nocturnal blood smear and an epidemiological investigation was conducted around all individuals with confirmed microfilaremia on nocturnal smear.

### Transmission assessment surveys

As recommended by the WHO, two post-treatment TAS were conducted 3 and 6 years after the last MDA for LF in 2009 in Togo. The first survey took place from March 26 to May 13, 2012 in the (then) 7 endemic districts (Tone, Kpendjal, Binah, Doufelgou, Kozah, Amou and Haho) grouped into four evaluation units (EU). As Tone district was subsequently split into 2 districts (Cinkassé and Tone) the second TAS was conducted from January 11 to 23, 2015 in 8 endemic districts grouped into the same four evaluation units [14].

In both TAS, school-going children aged 6–7 years old were selected using a cluster sampling method following the WHO guidelines. An immunochromatographic card test (ICT-Filariasis, Binax® Now, Scarborough, Maine, USA) using capillary blood was performed to detect filarial antigens from selected children. In 2015, ICT cards were used simultaneously with a filarial test strip (ICT-Filariasis, Alere® Now, Scarborough, Maine, USA) in two evaluation units: Cinkassé-Tone-Kpendjal EU and Binah-Doufelgou EU. In both TAS, a second ICT or filarial test strip (FTS) was performed for children who initially tested positive and a nocturnal blood sample was collected to prepare thick blood smears and dried blood samples on Whatman™ filter paper. The thick blood smears were stained with Giemsa stain and microscopy was performed for *W. bancrofti* microfilaria identification. Dried blood samples were tested using an enzyme-linked immunosorbent assay (TropBio ELISA Kit®, Townsville, Queensland, Australia) at the National Institute of Hygiene in Lomé (NIH) for detection of Og4C3 antigen [12, 13]. As with the ongoing surveillance system, individuals testing positive by Og4C3 ELISA and/or ICT/FTS were revisited and tested by nocturnal blood smear and an epidemiological investigation was conducted around all individuals with confirmed microfilaremia.

### Epidemiological investigation of microfilaremia-positive cases

The NLFEP conducted an epidemiological investigation around all confirmed microfilaremia cases from either the ongoing surveillance system or the TAS, according to an algorithm established in 2006 by the national programme [9]. The investigation consisted of screening for *W. bancrofti* microfilaremia among family members and neighbors to identify any active transmission in the community. The index case and any additional individuals with confirmed microfilaremia were treated yearly with albendazole and ivermectin for five years.

### Follow-up testing in 2016

In 2016, we attempted to trace all positive cases identified during the 2010–2015 surveillance period, whether positive by ICT card, FTS, Og4C3 ELISA, or nocturnal blood smear, to follow-up on their infection status. Each relocated case was tested simultaneously by FTS, Og4C3 ELISA and nocturnal blood smear.

### Data management and analysis

Laboratory-based surveillance data were recorded and analyzed using Microsoft Excel® (Microsoft, Redmond, Washington, United States). Transmission assessment survey data were entered and analyzed using EPI Info version 3.5.3 (Centers for Disease Control and Prevention, Atlanta, Georgia, USA). Maps were created using ArcGIS® 10.4 (Environmental Systems Research Institute, Inc., Redlands, California, USA).

## Results

Within the laboratory-based surveillance system, 26,581 blood smears were collected between 2010 and 2015. Over the same period, 6,788 dried blood samples were collected from the health facilities not equipped with a microscope. Microfilaremia and Og4C3 identification resulted in 1 (0.003%) and 19 (0.28%) positive cases respectively (Table 1). Nocturnal blood smears performed immediately after identification of each of the 19 Og4C3 positive cases were all microfilaremia negative. The one individual with a positive blood smear from the laboratory network was confirmed by a second microscopy. Overall, one case of microfilaremia was confirmed in the passive surveillance system, out of 33,369 individuals tested.

**Table 1:** Summary of the results of ongoing LF surveillance activities in Togo, 2010 to 2015

Sample type	Region	Year													Total
		2010		2011		2012		2013		2014		2015			
		*n*	Pos (%)	*n*	Pos (%)	*n*	Pos (%)	*n*	Pos (%)	*n*	Pos (%)	*n*	Pos (%)	*n*	Pos (%)
DBS	Savanes	271	2 (0.74)	271	0 (0)	273	1 (0.37)	328	1 (0.30)	378	1 (0.26)	350	0 (0)	1,871	5 (0.27)
DBS	Kara	351	1 (0.28)	327	0 (0)	340	0 (0)	330	0 (0)	330	1 (0.30)	330	0 (0)	2,008	2 (0.10)
DBS	Centrale	243	1 (0.41)	240	0 (0)	239	0 (0)	237	3 (1.27)	260	1 (0.38)	239	1 (0.42)	1,458	6 (0.41)
DBS	Plateaux	250	0 (0)	230	0 (0)	231	0 (0)	242	2 (0.83)	258	4 (1.55)	240	0 (0)	1,451	6 (0.41)
DBS	Sub-total	1115	4 (0.36)	1068	0 (0)	1083	1 (0.09)	1,137	6 (0.53)	1226	7 (0.57)	1,159	1 (0.09)	6,788	19 (0.28)
BS	Savanes	315	0 (0)	447	0 (0)	420	0 (0)	580	0 (0)	590	0 (0)	610	0 (0)	2,962	0 (0)
BS	Kara	471	0 (0)	826	1 (0.12)	961	0 (0)	940	0 (0)	930	0 (0)	941	0 (0)	5,069	1 (0.02)
BS	Centrale	538	0 (0)	563	0 (0)	580	0 (0)	560	0 (0)	573	0 (0)	580	0 (0)	3,394	0 (0)
BS	Plateaux	660	0 (0)	1173	0 (0)	1230	0 (0)	1655	0 (0)	1783	0 (0)	1748	0 (0)	8,249	0 (0)
BS	Maritime	357	0 (0)	709	0 (0)	789	0 (0)	915	0 (0)	820	0 (0)	761	0 (0)	4,351	0 (0)
BS	Lomé-Commune	94	0 (0)	375	0 (0)	510	0 (0)	480	0 (0)	530	0 (0)	570	0 (0)	2559	0 (0)
BS	Sub-total	2435	0 (0)	4093	1 (0.02)	4490	0 (0)	5130	0 (0)	5226	0 (0)	5210	0 (0)	26,584	1 (0)
Total		3550	4 (0.11)	5161	1 (0.02)	5573	1 (0.02)	6267	6 (0.10)	6452	7 (0.11)	6369	1 (0.02)	33,372	20 (0.06)

*Abbreviations*: *DBS* dried blood sample, *BS* blood smear, *n* number of samples collected, *Pos* number of positive cases, *(%)* percentage of positive cases

**Fig. 2:**
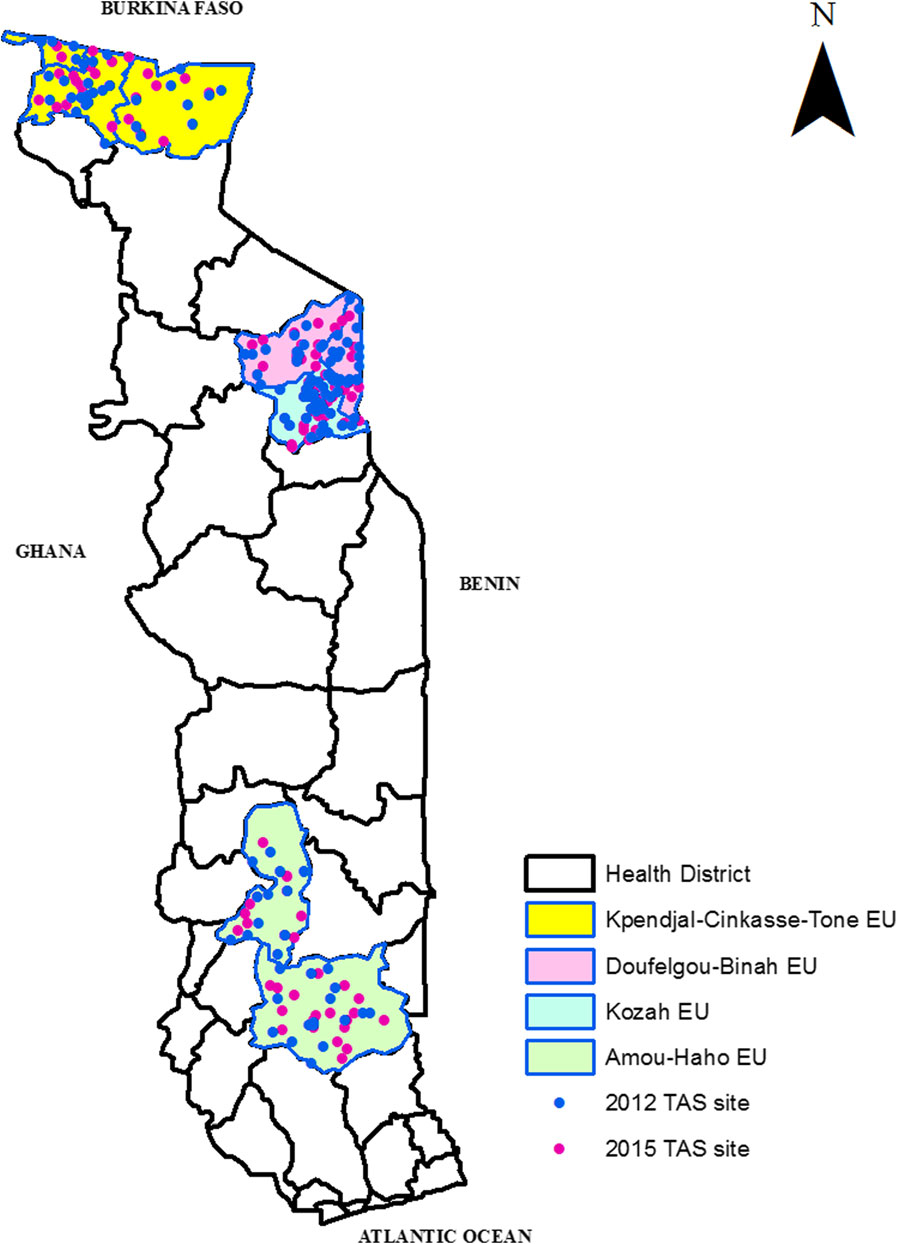
Transmission assessment survey sites in Togo, 2012 and 2015

For the TAS, 6380 children aged 6 to 7 years old in 131 schools and 6347 children in 124 schools were tested in 2012 and 2015 respectively (Fig. 2).

The numbers of ICT cards positive in 2012 and ICT card and/or FTS positive in 2015, were significantly below 18–20, the critical cut-off across all the EUs (Table 2). In 2012, all of the 13 ICT-positive cases were negative by ELISA and nocturnal microfilaremia. In 2015, however, three of the six ICT-positive cases in Kpendjal-Cinkassé-Tone EU were confirmed positive by Og4C3 and nocturnal microfilaremia.

**Table 2:** General characteristics and survey results from evaluation units in Togo, TAS 2012 and 2015

Year	EU	No. of districts	Population of the EU^a^	Total no. of children^b^	No. of schools in the EU	Data generated by SSB			No. of children tested	No. of schools surveyed	Sex ratio (M/F)	Positive ICT card or FTS *n* (%)	Positive mf and Og4C3
						No. of children to survey	No. of schools to survey	Critical cut-off					
2012	Kpendjal-Tone	2	462,946	46,627	457	1684	30	18	1706	31	860/846	8 (0.46)	0
2012	Doufelgou-Binah	2	153,686	11,786	201	1540	30	18	1549	30	763/786	1 (0.08)	0
2012	Kozah	1	234,228	10,645	235	1540	39	18	1550	40	747/803	0 (0)	na
2012	Amou-Haho	2	369,509	24,492	399	1556	30	18	1575	30	779/796	4 (0.25)	0
2015	Kpendjal-Cinkassé-Tone	3	426,578	47,122	499	1684	30	20	1701	30	916/785	6 (0.35)	3
2015	Doufelgou-Binah	2	169,860	12,220	218	1540	32	18	1542	34	789/758	0 (0)	na
2015	Kozah	1	259,115	13,712	237	1540	32	18	1547	32	770/772	0 (0)	na
2015	Amou-Haho	2	400,180	27,795	439	1556	30	18	1557	30	786/772	0 (0)	na

*Abbreviations*: *EU* evaluation unit, *na* not available, *M* male, *F* female, *mf* microfilaremia

^a^Data source: 2012 and 2015 population estimates by the Institut National de la Statistique et des Études Économiques et Démographiques (INSEED)

^b^First and second grade children aged 6 to 7 years

The epidemiologic investigation around the three microfilaremia confirmed cases (from Cinkassé and Tone IU of the 2015 TAS) yielded one additional microfilaria-positive case among the 35 family members and neighbors who were tested. Epidemiologic investigation of the single microfilariae positive case identified in 2011 through the routine PTS system detected no additional positive cases among the 252 family members and neighbours of the index case, but 11 (4.3%) cases of *Mansonella perstans* were found. All 5 confirmed cases detected during 2010–2015 post-treatment surveillance period (one by routine surveillance in 2011 and four through the 2015 TAS) were treated.

**Fig. 3:**
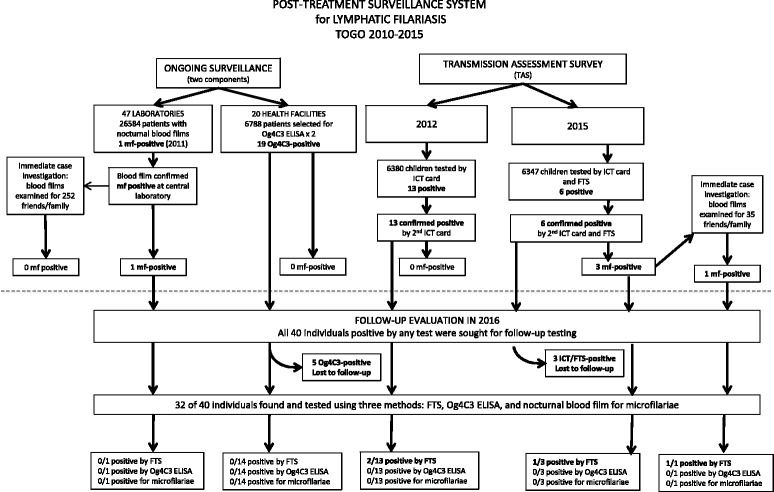
Post-treatment surveillance system for lymphatic filariasis in Togo, 2010–2015

In November 2016, we attempted to trace all 40 positive cases detected during the 2010–2015 PTS activities: 13 patients positive by ICT card during the 2012 TAS, 6 cases positive by ICT and/or FTS during the 2015 TAS, 1 microfilaria-positive case identified through the epidemiological investigation of a 2015 TAS case and 20 cases detected by the ongoing surveillance system (19 cases positive by Og4C3 ELISA, and one case positive for microfilaremia by the laboratory-based network). We located 32 (80%) of these 40 people and retested them using three methods simultaneously: FTS, Og4C3 ELISA and blood smear for nocturnal microfilaremia. Four of 32 individuals (12.5%) were remained FTS positive: two in Tone District from the 2012 TAS and two in Cinkassé district from the 2015 TAS. No one tested positive for microfilaremia or Og4C3 antigen (Table 3, Fig. 3).

**Table 3:** Results of the 2016 follow-up investigation of cases identified during post-treatment surveillance

PTS activity	Region	District	Location	PTS positive cases (number)	Age of positive cases (years)	Sex of positive cases	Year positive cases detected	2016 follow-up investigation			
								Lost to follow-up	Positive FTS	Positive Og4C3	Positive mf
Og4C3 ELISA follow-up											
Og4C3 ELISA	Plateaux	Amou	Game	6	13	F	2013	Y	na	na	na
					28	M	2013	N	N	N	N
					25	F	2014	N	N	N	N
					35	M	2014	N	N	N	N
					27	F	2014	N	N	N	N
					34	F	2014	N	N	N	N
Og4C3 ELISA	Centrale	Blitta	Assoukoko	2	19	M	2013	N	N	N	N
					42	M	2014	N	N	N	N
Og4C3 ELISA	Centrale	Sotouboua	Boulohou	1	31	F	2013	N	N	N	N
Og4C3 ELISA	Centrale	Sotouboua	Djarkpanga	3	67	M	2010	Y(D)	na	na	na
					40	F	2013	Y	na	na	na
					41	M	2015	N	N	N	N
Og4C3 ELISA	Kara	Dankpen	Koulfiekou	1	37	M	2014	N	N	N	N
Og4C3 ELISA	Kara	Keran	Koutougou	1	55	M	2010	N	N	N	N
Og4C3 ELISA	Savanes	Kpendjal	Pogno	2	22	F	2010	Y	na	na	na
					48	M	2014	Y	na	na	na
Og4C3 ELISA	Savanes	Oti	Gando	1	60	M	2013	N	N	N	N
Og4C3 ELISA	Savanes	Oti	Tchamonga	1	42	M	2012	N	N	N	N
Og4C3 ELISA	Savanes	Tandjouare	Doukpelou	1	41	M	2010	N	N	N	N
Laboratory microscopy follow-up											
Microscopy	Plateaux	Est-Mono	Atikpai	1	37		2011	N	N	N	N
TAS follow-up											
2012 TAS	Savanes	Tone	Nansongue	5	6	M	2012	N	P	N	N
					6	M	2012	N	N	N	N
					7	F	2012	N	N	N	N
					7	F	2012	N	N	N	N
					7	M	2012	N	N	N	N
2012 TAS	Savanes	Tone	Korbongou	2	6	M	2012	N	N	N	N
					7	F	2012	N	P	N	N
2012 TAS	Savanes	Cinkasse	Cinkasse	1	6	M	2012	N	N	N	N
2012 TAS	Kara	Doufelgou	Tabitanta	1	7	M	2012	N	N	N	N
2012 TAS	Plateaux	Haho	Azakpe	4	6	F	2012	N	N	N	N
					6	M	2012	N	N	N	N
					6	F	2012	N	N	N	N
					6	F	2012	N	N	N	N
2015 TAS	Savanes	Cinkasse	Kourientre	2^a^	6	F	2015	N	N	N	N
					35	F	2015	N	P	N	N
2015 TAS	Savanes	Cinkasse	Timbou	1	7	M	2015	N	P	N	N
2015 TAS	Savanes	Kpendjal	Pogno	1	6	M	2015	N	N	N	N
2015 TAS	Savanes	Tone	Kambere	3	6	M	2015	Y	na	na	na
					7	M	2015	Y	na	na	na
					7	M	2015	Y	na	na	na
Total				40				8	4	0	0

^a^Including one mother found positive while investigating a positive child from the 2015 TAS

*Abbreviations*: *N* negative/no, *Y* yes, *P* positive, *na* not applicable, *D* deceased, *F* female, *M* male, *mf* microfilaremia

## Discussion

The sustained success of LF elimination depends on a careful and comprehensive surveillance system to detect potential post-MDA recrudescence, particularly in high-risk areas such as districts sharing borders with neighbouring countries where LF remains endemic. The surveillance should include epidemiological investigation around microfilaria-positive cases to exclude potential hotspots. Following the cessation of MDA for LF, the WHO recommends conducting five years of post-treatment surveillance consisting of two TAS in the implementation units where the treatment is stopped. The WHO has developed clear guidelines for conducting the surveys. In addition, the WHO suggests screening for microfilariae as well as serologic markers of LF in target populations such as the army, students and blood donors [10]. Entomologic surveillance (xenomonitoring) is also indicated in the post-intervention surveillance phase to detect infection in the mosquito vector, which is a sign of persisting transmission. The lack of standard operating procedures to conduct these additional surveillance activities is a serious challenge for the implementation of PTS, which is now limited to only two TAS in many of LF-endemic countries.

To address gap in guidance, and given the focal nature of LF in Togo, the management of the NLFEP saw a need to develop a PTS system that went beyond the two recommended TAS. This was a countrywide ongoing surveillance system that covered not only districts identified as endemic, but also those classified as non-endemic during the baseline mapping surveys [7]. An evaluation in 2010 revealed that the surveillance system was capable of identifying positive cases [11]. Overall, 322 data collection points consisting of laboratories, peripheral health facilities and schools were distributed across the country, with more than 33,000 people examined over six years of surveillance during which 5 microfilaria-positive cases were detected. One of the microfilaria-positive individuals identified through the ongoing surveillance system was from a non-endemic district, highlighting the need for establishing a countrywide surveillance system that includes endemic and non-endemic districts.

A critical component of this surveillance system was the in-depth epidemiological investigation around each microfilaria-positive case. Identification and testing of neighbours and relatives demonstrated that the one microfilaremia-positive person identified through the ongoing surveillance represented an isolated case. Investigation of the cluster of three positive cases from the 2015 TAS turned up one additional microfilaremia-positive person, but an evaluation of the travel histories of these individuals revealed that all 4 immigrated together to Togo in 2011 from Cote d'Ivoire, where LF is endemic and MDA was covering only 31% of the 74 endemic districts [15]. The absence of additional cases in neighbours and family indicated that local transmission was not occurring. These four positive cases were located in the Savanes Region, where populations frequently move across borders for economic and social reasons [16, 17]. While it is believed that areas from which filariasis transmission has been interrupted are not likely to have infection re-introduced from neighboring uncontrolled areas [18, 19], the possibility of importation of LF does exist and must be considered in establishing a surveillance system. This is especially important for countries such as Togo and Cambodia that have been declared free of LF but receive many visitors and migrants from neighboring countries with active LF transmission [20]. Future surveillance and research activities in Togo must therefore focus on migrant populations as well as on communities at the borders in order to ensure that these do not become potential transmission reservoirs that could lead to the resurgence of LF. However, the regular countrywide distribution of long lasting impregnated nets for malaria control could also prevent recrudescence of LF transmission since both diseases are transmitted by *Anopheles* vectors in Togo [21–23].

During the PTS, four detection methods were employed: the ICT card, FTS and Og4C3 ELISA for the detection of *W. bancrofti* circulating antigen, and nocturnal blood smears for the detection of microfilariae by microscopy. In the literature one study found that Og4C3 ELISA is more sensitive than the ICT while another showed that FTS is more sensitive than the ICT [24–26]. Circulating filarial antigen (CFA) can persist in microfilaria-negative patients for at least three years following treatment due to its slow clearance [27, 28]. This likely explains the presence of antigen in 2 of our 5 previously microfilaria-positive patients when they were tested in 2016, as well as persistence of antigen in two ICT-positive individuals from the 2012 TAS.

One additional challenge of PTS is the documented poor sensitivity of the nocturnal blood smear for detecting parasites in individuals with low microfilarial load [29]. Among those patients who tested positive by ICT or Og4C3 ELISA but negative by nocturnal blood smear, more sensitive methods for detecting microfilariae, such as the nucleopore filtration or concentration methods would have provided more definitive information on the microfilarial status of these CFA-positive/microfilaria-negative individuals. Blood concentration methods should be considered in future surveillance activities as an additional step to confirm the absence of microfilariae in CFA positive individuals with a negative nocturnal blood smear.

## Conclusions

In addition to the two post-MDA surveys recommended by the WHO in LF-endemic districts, Togo conducted six years of supplementary surveillance that covered the entire country, including both endemic and non-endemic districts. For six years following the cessation of the MDA the system did not detect any evidence of recrudescence or hotspot. In addition, the long-term follow-up of 32 of the 40 antigen or microfilaria-positive cases identified during this PTS demonstrated resolution of the antigenemia in 28 of 32 cases. Epidemiological investigation of all microfilaria-positive cases was a critical component of this surveillance system and must be encouraged in other countries conducting post-MDA surveillance to help detect new transmission clusters so that appropriate and timely actions are taken to prevent the possible resurgence of the disease. The system implemented in Togo was robust enough to provide critical evidence to inform the validation dossier submitted to WHO and prove the successful elimination of LF as a public health problem in Togo.

## Abbreviations

CFA: Circulating filariasis antigen
EU: Evaluation unit
FTS: Filariasis test strip
ICT: Immunochromatographic card test
IU: Implementation unit
LF: Lymphatic filariasis
MDA: Mass drug administration
NIH: National Institute of Hygiene
NLFEP: National Lymphatic Filariasis Elimination Programme
PTS: Post-treatment surveillance
TAS: Transmission assessment survey
WHO: World Health Organization
